# Novel Surface-Enhanced Raman Spectroscopy Techniques for DNA, Protein and Drug Detection

**DOI:** 10.3390/s19071712

**Published:** 2019-04-10

**Authors:** Chuanpin Chen, Wenfang Liu, Sanping Tian, Tingting Hong

**Affiliations:** School of Pharmaceutical Sciences, Central South University, Changsha 410013, Hunan, China; chuanpin.chen@csu.edu.cn (C.C.); liuwenfang@csu.edu.cn (W.L.); 1152349979@qq.com (S.T.)

**Keywords:** DNA, drug, protein, Surface-enhanced Raman spectroscopy

## Abstract

Surface-enhanced Raman spectroscopy (SERS) is a vibrational spectroscopic technique in which the Raman scattering signal strength of molecules, absorbed by rough metals or the surface of nanoparticles, experiences an exponential growth (10^3^–10^6^ times and even 10^14^–10^15^ times) because of electromagnetic or chemical enhancements. Nowadays, SERS has attracted tremendous attention in the field of analytical chemistry due to its specific advantages, including high selectivity, rich informative spectral properties, nondestructive testing, and the prominent multiplexing capabilities of Raman spectroscopy. In this review, we present the applications of state-of-the-art SERS for the detection of DNA, proteins and drugs. Moreover, we focus on highlighting the merits and mechanisms of achieving enhanced SERS signals for food safety and clinical treatment. The machine learning techniques, combined with SERS detection, are also indicated herein. This review concludes with recommendations for future studies on the development of SERS.

## 1. Introduction

Raman spectroscopy, as molecular vibration-based technique, is used to study molecular structures based on the Raman scattering effect, discovered by C.V Raman in 1928. However, for conventional Raman spectroscopy, the relatively weak Raman scattering process, with an incident intensity of 10^−10^ times, reduces the sensitivity and limits the range of usage. Therefore, it is necessary to utilize the enhancement effect of material surfaces for Raman studies. In 1974, Fleischmann et al. measured pyridine molecules adsorbed on the surface of roughed silver electrodes, and it was found that the Raman scattering signal intensity was increased by 10^6^ times [1]. Afterwards, Van Duyne verified this phenomenon and called it a surface enhancement effect [2]. The enhancement factor (EF) of Surface-enhanced Raman spectroscopy (SERS) can reach as much as 10^10^, with resonance, facilitating the development of an efficient analytical tool for detecting low-concentration analytes. With the development of SERS, the low sensitivity of Raman spectroscopy can be improved, and the structural information, which is difficult to detect with conventional Raman spectroscopy, can also be obtained [3,4,5,6,7]. 

Electromagnetic (EM) theory suggests that the EF of SERS mainly depends on the characteristics of SERS substrates [8,9]. During the analysis process, the analytes are adsorbed on an active SERS substrate, and monochromatic radiation from a laser is applied to irradiate the substrate. The resulting scattering can be analyzed via a Raman spectrometer. For practical applications, it is necessary to choose an effective substrate that can offer reasonable enhancement, be reproduced and be reasonably robust. Among various metals, gold and silver are commonly used to develop the substrates. With in-depth research on nanomaterials and nanotechnology, much more attention has been paid to designing desirable SERS substrates to generate hot spots. This research indicates that enhancing the local field of hot spots between two or multiple nanoparticles can significantly magnify Raman signals. Due to the improved detection efficiency, SERS has been widely utilized to identify a variety of analytes, such as DNA fragments, drugs of abuse, and proteins [10,11,12,13,14,15]. Conventional DNA analysis strategies usually involve complicated analytical procedures and suffer from a high cost. Recently, SERS has become a promising technique for the rapid recognition and structural characterization of DNA, offering an ultrahigh sensitivity and detailed fingerprint information. Since diagnostic tests play an important role in disease detection, developing a sensitive and specific analytical tool to measure multiple protein markers simultaneously has gained tremendous attention [16,17,18]. Meanwhile, SERS presents a satisfactory technique for multiplexed assays. Combined with chemometric methods, SERS has a desirable resolution ability, analyzing and classifying complex spectral data. Due to its unique advantages in drug detection, SERS also exhibits a powerful potential for drug safety and medication guides.

Cailletaud et al. described recent advances in the applications of SERS in pharmaceutical analysis [19]. In the present review, we will summarize the developments of SERS utilized in the field of analysis, focusing on three aspects, including DNA, protein and drug detection. Herein, innovative methods developed for the detection of microbe/disease-related DNA targets for food safety and clinical diagnosis will be summarized. The application of SERS in the detection of protein in the blood and disease-related protein markers will also be discussed in detail. In addition, we will review the employment of a state-of-the-art SERS-based technique for drug detection. The advantages of applying SERS for DNA, protein and drug detection will be highlighted in this article.

## 2. SERS for DNA Detection

DNA, the carrier of genetic information, can form genetic instructions and guide the biological development and functional operation of life. To investigate the nature of life, much more attention has been paid to genetic detection due to its important significance in the early diagnosis and prevention of disease [20,21,22,23]. Nowadays, SERS has been successfully used to identify DNA sequences, distinguish DNA from RNA in a mixture and monitor the hybridization of individual DNA in microfluidics [24,25,26,27,28,29,30,31,32,33,34]. We review the applications of SERS for DNA detection in the following sections.

### 2.1. SERS in the Detection of Microbe-Related DNA Targets for Food Safety

#### 2.1.1. Sandwich Detection Method

**Figure 1:**
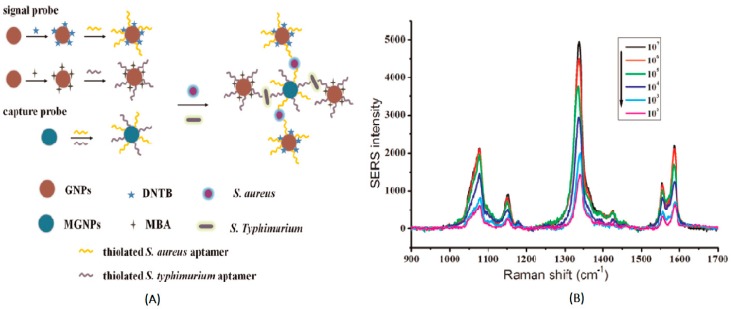
(**A**) Schematic illustration of the aptasensor, immobilized on Fe_3_O_4_ magnetic Au nanoparticles (MGNPs) for the simultaneous detection of *S. aureus* and *S. typhimurium*, based on Au nanoparticles (GNPs) enhanced by Raman intensity. (**B**) Raman scattering spectra of different concentration of *S. aureus* and *S. typhimurium* (10^2^ cfu mL^−1^, 10^3^ cfu mL^−1^, 10^4^ cfu mL^−1^, 10^5^ cfu mL^−1^, 10^6^ cfu mL^−1^, and 10^7^ cfu mL^−1^) (Zhang et al. [36]).

In the sandwich detection method, the capture probes (nanoparticles labeled with complementary DNA sequences) and signal probes (DNA-functionalized nanoparticles labeled with Raman reporter molecules) are designed [35]. The two DNA sequences are non-complementary, but both of them are complementary to the target DNA. When the target DNA is introduced, hybridization between the target DNA and probes results in the formation of a sandwich structure and the aggregation of the nanoparticles, based on the complementation pairing rule. In this process, the concentration of hot-spot formation is significantly increased, and the Raman signal of Raman reporters is greatly enhanced. The SERS-based sandwich detection method provides a special hybridization method for detecting DNA, with satisfactory selectivity and stability. The multiple detection of target DNA, as a trend in SERS detection, is much more meaningful in reality because of its timesaving advantage. In order to increase the specificity of DNA and Raman reporters on the probe, it is necessary to avoid overlapping characteristic signals between Raman reporters. Zhang et al. used 5,5′-dithiobis(2-nitrobenzoic acid) (DNTB) and mercaptobenzoic acid (MBA) as Raman reporters to simultaneously measure the staphylococci *S. aureus* and *S. typhimurium* in pork, with detection limits of 35 cfu/mL and 15 cfu/mL, respectively (Figure 1) [36]. Herein, gold nanoparticles were modified with MBA, DNTB and aptamer to develop a signal probe, and an enhanced Raman intensity was obtained due to the utilization of nanoparticles. Meanwhile, Fe_3_O_4_ magnetic gold nanoparticles were immobilized with aptamers to prepare the capture probe. As single strand nucleic acids, which can normally form three-dimensional conformations, aptamers exhibited a high affinity with the target molecules. The specificity of this method used for detecting *S. aureus* and *S. typhimurium* was investigated. This indicated that the signal intensities of other bacteria, including *Escherichia coli*, *Shigella dysenteriae*, *Vibrio parahaemolyticus* and *Bacillus cereus*, were much lower than those of the *S. aureus* and *S. typhimurium*. This could be attributed to the high affinity and specificity of the aptamer with its target, facilitating the accomplishment of sandwich detection. 

#### 2.1.2. Amplification Method

For low-content DNA detection, the hot spots generated in the sandwich detection method are not sufficient to obtain detectable Raman signals. In order to overcome this disadvantage, the amplification method is investigated [37].

The amplification method can be divided into two categories: the product expansion and signal amplification methods. The nature of the amplification method is based on DNA hybridization. Product expansion is mainly based on the polymerase chain reaction (PCR). PCR, a special process of DNA replication, can amplify specific DNA fragments and then sharply increase their content. SERS can determine the existence of the target DNA (tDNA) sequence by detecting PCR products. Isola et al. used SERS-active labels as primers to amplify related HIV genes by PCR, and the applied SERS detected Raman labels to determine tDNA [38]. The results showed that the SERS-based PCR can effectively improve the sensitivity and selectivity of detection.

In the signal amplification method, tDNA is used to trigger the hybridization in the long chains between the specific structure of the DNA templates, immobilized on the substrate, and Raman labeled primers fixed on nanoparticles. The long chains, containing lots of Raman labeled primers, are obtained at the same time. Using this method, *Bacillus thuringiensis* (Bt) transgenic sequence was measured with an limit of detection (LOD) of 50 pM (S/N = 3) [39]. To evaluate the selectivity of the proposed method for detecting the Bt transgenic gene fragment (tDNA-Bt), a haipin DNA (H3) was introduced to develop the biosensing platform. In this system, tDNA-Bt could hybridize with the complementary sequences of the additional H3, resulting in DNA nanowires. Subsequently, gold nanoparticle probes grew along the nanowires due to the strong affinity with the biotin-streptavidin system. Herein, gold nanoparticle probes could form hot spots between particles, and a strong SERS signal was obtained. This suggested that the detection of tDNA was not influenced by the addition of H3. In this work, the accuracy and sensitivity of the proposed SERS-based method was comparable with real-time PCR. The 35S promoter gene, a marker of a genetically modified organism, has been detected by SERS-based rolling circle amplification, with an LOD of 6.3 fM and detection range from 100 fM to 100 nM [40]. The rolling circle amplification (RCA) reaction was utilized to develop an RCA-SERS sandwich assay for enhancing the SERS spectra of target molecules. Herein, an oligonucleotide probe was immobilized on a gold slide, and 5,5′-dithiobis(2-nitrobenzoic acid) (DTNB) was applied to form a self-assembled monolayer on gold nanorods. The concentration of the target was determined by the SERS spectra of DTNB on the nanorods. In a traditional SERS sandwich assay, two target probes are attached to rod-shaped gold nanoparticles and a gold slide. Then, they are hybridized with the target oligonucleotides. Compared with the conventional SERS sandwich method, the RCA-SERS strategy exhibited an improved sensitivity, and amplification occurred after the first hybridization, with the probe on the target sequence and gold slide.

In order to obtain enhanced or amplified SERS signals, the product expansion and signal amplification methods were both used to detect amplification products. Nevertheless, the difference between them is that the former method amplifies the number of DNA fragments, and the later one amplifies the length of DNA fragments. The amplification method has attracted much more interest due to its flexibility in design and high sensitivity in detection. It provides a promising application prospect for DNA detection.

Detecting the microbes in food is important for guaranteeing the quality and safety of food, avoiding the occurrence of food poisoning. A traditional microbial test is usually time-consuming, with a long separation and cultivation process. In comparison, SERS can recognize the microbe by detecting the related gene sequences in less time, with a high specificity and accuracy. 

### 2.2. SERS in the Detection of Disease-Related DNA Targets for Clinical Diagnosis

The mutation or methylation of genes may lead to diseases. Nucleic acids, a type of important cancer biomarker, can be used for disease diagnosis [41,42,43]. In this section, we summarize recent applications of SERS for disease-related DNA target detection in clinical diagnosis.

#### 2.2.1. DNA Hybridization Method

**Figure 2:**
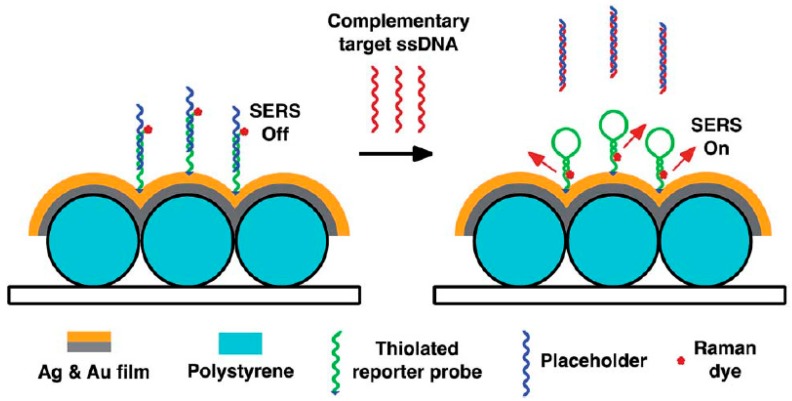
DNA bioassay-on-chip detection scheme (Ngo et al. [47]). SERS: Surface-enhanced Raman spectroscopy.

In the DNA hybridization method, Raman-labeled ssDNA is fixed on the nanoparticles as probes. After introducing the target DNA, hybridization between the probes and the target DNA will change the intensity of Raman molecules by changing the distance between the nanoparticles and Raman molecules. The identification of the target DNA and detection of its content can be obtained by measuring the intensity changes of the Raman molecules [44,45,46]. Based on the above mechanism, dengue diagnosis and HIV-1 detection have been successfully achieved (Figure 2) [47,48]. 

A homogeneous DNA bioassay-on-chip system was fabricated by utilizing SERS detection on a bimetallic nanowave chip. Since SERS enhancement could decrease with an increase in the Raman dye-metal surface distance, the Raman dye-chip’s surface distance presented low SERS signals. The complementary target ssDNA was hybridized with the placeholders, and then the reporter probes facilitated the formation of hairpin structures. This design enabled the achievement of strong SERS signals, because Raman dyes were introduced close to the nanowave chip’s metal surface. Additional Raman active molecules need to be introduced, although this method is flexible and rich in designing systems. This bioassay-on-chip platform provided an efficient method for detecting the nucleic acid sequence of dengue in point-of-care clinical diagnosis applications. 

#### 2.2.2. Asymmetry Signal Amplification Method

The SERS-based amplification method has a high sensitivity, specificity and accuracy. This method exhibits satisfactory results in microbial detection in food as well as in clinical diagnosis. The single base extension reaction-based SERS had a good result in detecting methylated DNA, with an LOD of 3pM, and it could even distinguish a methylation level as low as 1% in the tumor suppressor gene, CDKN2/p16/MTS1 (p16), from the mixtures [49]. Traditional methods used for the DNA methylation assay were time-consuming and laborious. Herein, a simple and sensitive DNA methylation assay based on SERS and a single base extension reaction was developed. Because of the introduction of methylated DNA, gold nanoparticle-functionalized capture probes could attach to a cyanine 5-deoxyribonucleoside triphosphate by a single base extension reaction. 

**Figure 3:**
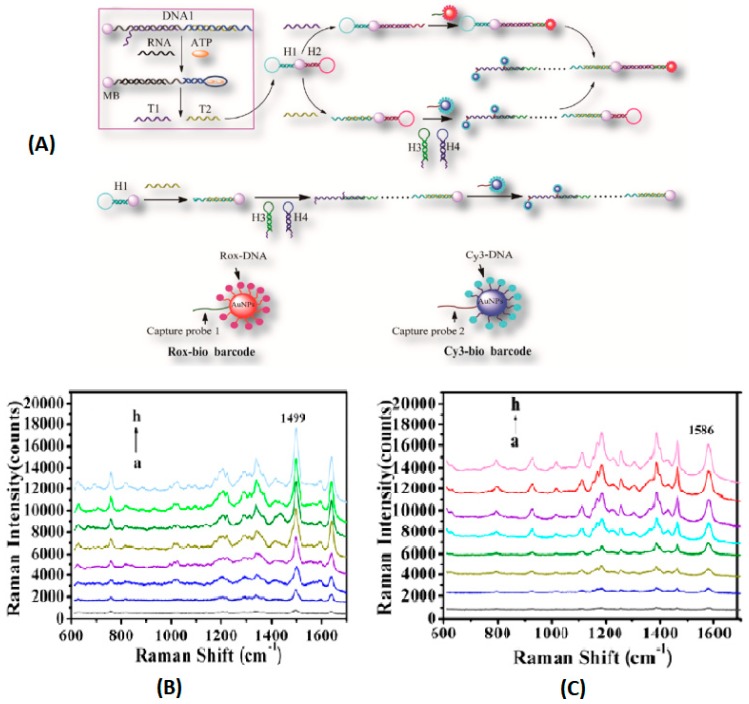
(**A**) Illustration of the asymmetric signal amplification SERS assay and process of HCR. (**B**) SERS spectra for increasing concentrations of ATP (a, 0; b, 1.0 × 10^−7^; c, 5.0 × 10^−7^; d, 1.0 × 10^−6^; e, 5.0 × 10^−6^; f, 1.0 × 10^−5^; g, 5.0 × 10^−4^; h, 1.0 × 10^−4^ M). (**C**) SERS spectra for increasing concentrations of miR-203 (a, 0; b, 1.0 × 10^−15^; c, 5.0 × 10^−15^; d, 1.0 × 10^−14^; e, 5.0 × 10^−14^; f, 1.0 × 10^−13^; g, 5.0 × 10^−13^; h, 1.0 × 10^−12^ M). (Ye et al. [50]).

The local electromagnetic field increased after the further addition of gold nanoparticles, and a high SERS signal was obtained. However, no SERS signal was observed when using unmethylated DNA. Compared with the gold nanoparticle-based colorimetric assay and microarray-based methylation sensitive single nucleotide primer extension assay, the sensitivity of this method was enhanced by five and two orders of magnitude. The results indicated that the single base extension reaction-based SERS offered a desirable strategy for the DNA methylation assay, owing to the initial utilization of a bisulfite treatment, and this platform might be applied to monitor the methylation status in tumor-linked genes for cancer diagnosis. The above amplification method was only suitable for a type of molecule detection. For the sake of achieving the simultaneous detection of multiple biomarkers with different levels, the asymmetric signal amplification method was explored [50]. Herein, the asymmetric signal amplification was initiated by the assembled bifunctional probe. The quadratic signal amplification mode responds to low-concentration markers, and the linear amplification mode corresponds to the high-concentration markers (Figure 3). By using the combined bio-barcode probe and hybridization chain reaction amplification method, the LODs of microRNA and ATP were 0.15 fM and 20 nM, respectively. This method provided an efficient method for the simultaneous detection of various biomarkers with significantly different levels and an improved detection sensitivity.

#### 2.2.3. Gene Chips Method

A gene chip, SERS-based DNA array/sensor was used to detect pathogens or biomarkers for fast and sensitive disease diagnosis. A pattern formed by multiple Au nanowire sensors was developed for the multiplex sensing of the target DNA [51]. Au nanowire and Au NPs have gained tremendous attention in relation to their application in SERS-active platforms due to their well-defined geometry and superb physicochemical properties. Herein, Au nanowires on the substrate were incubated with the target DNA. Subsequently, an Au particle-on-wire structure was prepared by the sandwich hybridization of probe-target-reporter DNA. The resulting Au particle-on-wire structure could create SERS hot spots in the gaps between nanowires and nanoparticles, when the target DNA possessed sequences complementary to the reporter DNA and the probe DNA. This system was operated by the self-assembly of Au NPs onto Au nanowire in the presence of target DNA, and the particle-on-wire sensors could generate reproducible SERS signals only in proportion to a DNA concentration ranging from 10 pM to 10 nM. A SERS-based assay was also applied to detect bacterial meningitis pathogens [52]. Before the SERS assay, bacterial meningitis pathogen DNA, extracted from patient clinical samples, was amplified by PCR. In this study, nine clinical samples presented a satisfactory discrimination, which facilitated the identification of the pathogen via SERS. Furthermore, various infectious diseases could be detected by changing the sequences of the reporter and capture probes. This indicated that SERS combined with a gene chip could achieve multiplex detection and quantitative detection due to its high sensitivity, accuracy and reproducibility. A gene chip is a promising choice for future development directions, though it is mainly used in laboratories nowadays due to its high cost [53,54].

A tissue slice sample can be utilized to obtain accurate information about patients for liver cancer diagnosis. Nevertheless, the employment of a Raman spectrum in diagnosing liver cancer is mainly limited to pure liver cell lines, and few researches focused on applying SERS for liver cancer and normal tissue slices. Chen et al. applied the SERS technique in investigating the tissue sections of cancerous and normal livers, obtaining information on the changes of biological composition in the tissues [55]. Herein, silver nanoparticles were added to improve the spectral signal of tissue slices due to the specific physical and chemical characteristics of Ag-NPs. Combined with the principal component analysis (PCA) and linear discriminant analysis (LDA), SERS presented a favorable resolution ability for cancerous and normal liver tissues by acquiring complex spectral data. It was found that the proportion of DNA in the liver cancer group was higher than that in the normal group. The PCA-LDA method was utilized to analyze the sensitivity and specificity of the diagnosis. The results indicated that the fingerprint SERS spectra could discriminate normal and cancerous tissues, presenting a promising potential in the clinical detection of liver cancer.

In addition to the field of food safety and clinical diagnosis, SERS can also be used to detect DNA in other areas, such as the study of the interaction between DNA and small drug molecules or other exogenous substances at the molecular level. The SERS-based assay displayed an important theoretical significance in understanding the interaction mechanism of DNA and exogenous agents, including a chemotherapeutic drug (cisplatin), an organic dye (methylene blue) and a metal ion (Hg^II^) [56]. Herein, binding information was presented by the specific and characteristic vibrational alterations of the SERS spectra in Table 1.

## 3. SERS for Protein Detection

Proteins are the basis of life and the undertakers of life activities. Some simple proteins can be determined by using SERS to identify the amino acid residues or polypeptide skeleton structure [57,58]. Moreover, the SERS-based antigen-antibody reaction can identify some functional proteins [59,60]. SERS for protein detection is mainly used in clinical diagnosis by detecting specific disease-related protein biomarkers. 

The death rate of cancer is very high, since people usually realize that they are suffering from cancer in the middle-late stage of their lives, when it is already hard to cure [61,62,63]. There are few differences between normal people and cancer sufferers on some special proteins, such as the relative content and conformation. These differences can be used for diagnosing cancers. Protein, as an important cancer biomarker, is expressed in cancerous parts in the early stages [64,65,66,67]. However, conventional methods have difficulty detecting the proteins at low levels. The SERS-based immunoassay has the potential to detect protein biomarkers and be used in early diagnosis and postoperative detection due to its high sensitivity. It is a win-win result of saving resources and improving the survival rate of patients.

### 3.1. SERS in the Detection of Protein in the Blood for Clinical Diagnosis

Cervo, S et al. investigated the application of SERS for detecting ‘luminal A’ breast cancer at different stages [68]. It was found that the serum of patients presented a higher Raman peak intensity than the serum of normal people, at 721, 1093, 1324, 1444 cm^−1^, using the Raman spectra combined with PCA. Compared with the gold standard method mammography, recently utilized for screening processes, SERS spectroscopy, associated with the multivariate data analysis method, exhibited a promising potential in discriminating healthy subjects from breast cancer samples, with a high sensitivity. Moreover, compared with other diagnostic methods, SERS displayed specific merits, such as a minimally invasive application and a fast and portable operation. Non-invasive nasopharyngeal cancer detection was developed by mixing silver nanoparticles with blood plasma to enhance the Raman scattering signals of biomolecules [69]. PCA and LDA were employed to analyze and classify the blood plasma SERS spectra, obtained from cancer patients and health subjects. High diagnostic sensitivity (90.7%) and specificity (100%) were achieved in this study. Another noninvasive cancer detection strategy was explored by combing membrane electrophoresis and silver nanoparticle-based SERS [70]. First, albumin and globulin proteins were isolated from blood plasma via membrane electrophoresis. Subsequently, silver nanoparticles were mixed with the samples to perform the SERS detection process. In comparison with the direct SERS analysis of untreated blood plasma, the proposed method could reduce the spectral variability due to the elimination of exogenous substances and the highly variable plasma constituents, except for the target proteins. As for the untreated samples, there was some overlap between the patients and normal groups. Nevertheless, the PCA results indicated that the data points for the normal group and cancer groups could form completely separated clusters, with a 100% diagnostic sensitivity and specificity. Furthermore, to avoid the accidental situation caused by a small sample and guarantee the accuracy and universality of the test results, it is necessary to enlarge the sample size. PCA combined with SERS was also used to identify ricin B chain in blood [71]. Ricin B chain (RBC) is a lectin that attaches to galactose residues on the cell surface. In this study, aptamer-funtionalized silver film-over-nanosphere (AgFON) substrate was used to obtain stable SERS enhancement factors in human blood. The PCA of the SERS spectra could distinguish the AgFONs exposed to RBC from those without RBC exposure. This work provided an efficient platform for detecting and removing ricin from contaminated blood. 

### 3.2. SERS in the Detection of Disease-Related Protein Markers for Clinical Diagnosis

**Figure 4:**
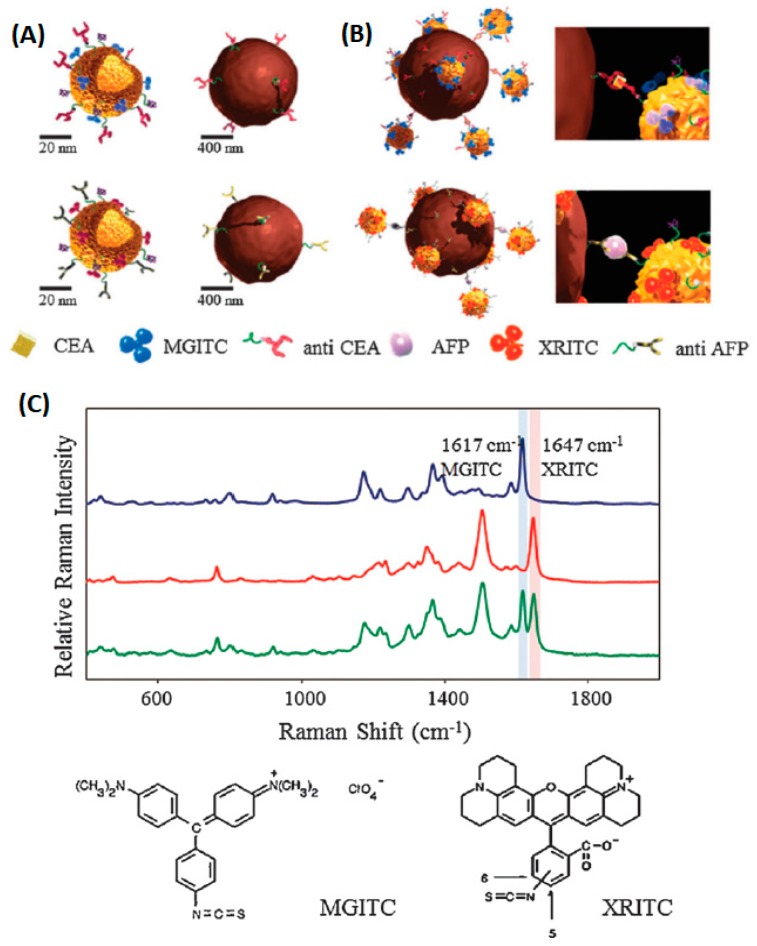
Schematic of the SERS-based duplex immunoassay using hollow gold nanospheres (HGNs) and magnetic beads. (**A**) Conjugation of malachite green isothiocyanates (MGITCs) (Raman reporter) and carcinoembryonic antigen (CEA) antibodies to HGNs (upper left), conjugation of X-rhodamine-5-(and-6)-isothiocyanate (XRITCs) (Raman reporter) and α-fetoprotein (AFP) antibodies to HGNs (upper right), anti-CEA immobilized magnetic beads (lower left), and anti-AFP modified magnetic beads (lower right). (**B**) Formation of sandwich immunocomplexes between HGNs and magnetic beads for CEA and AFP. (**C**) Raman spectra of HGN probes labeled with MGITC (blue), XRITC (red) and a 1:1 mixture of HGNMG and HGNXR (green) (Chon et al. [76]).

**Figure 5:**
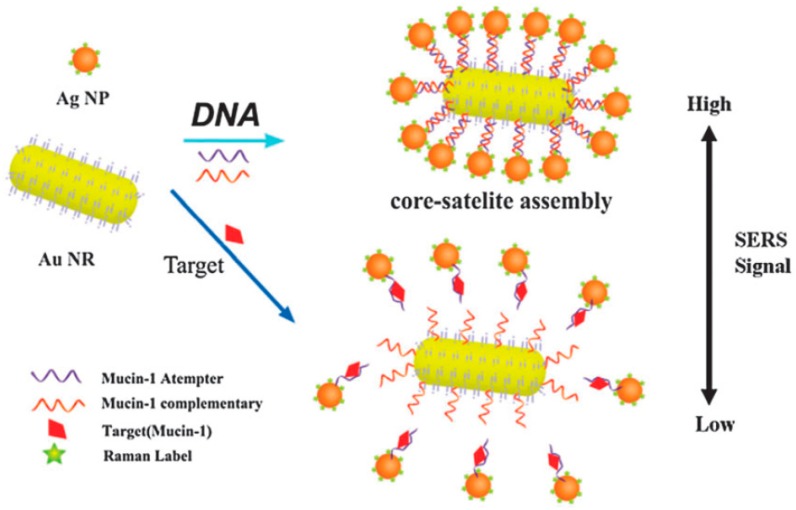
Scheme of the SERS aptasensor for the detection of Mucin-1 based on Au NRs-Ag NP core-satellite assemblies (Feng et al. [78]).

The immunoassay is based on specific the identification and hybridization of the antibody and antigen. The process is similar to DNA hybridization [72,73,74]. The SERS-based immunoassay successfully identified tumor cancer cell protein markers [75,76,77]. As can be seen in Figure 4, functionalized hollow gold nanospheres and magnetic beads were utilized to develop the SERS-based immunoassay. Herein, the carcinoembryonic antigen (CEA) and α-fetoprotein (AFP) were selected as target marker proteins. The sandwich-type immunocomplexes between the hollow gold nanospheres and magnetic beads were formed for CEA and AFP. Nevertheless, the hollow gold nanospheres and magnetic beads did not form a sandwich complex in the absence of the antigen. The dual cancer markers in blood serum could be detected simultaneously under a single excitation wavelength The aptamer recognition method for protein detection is based on the bonding mechanism of protein and aptamer. A novel core-satellite structure was constructed by using DNA as the linker (Figure 5). 

In this method, aptamer was fixed on the nanoparticles as capture probes, and nanoparticle-based aptamer complementary DNA fragments and Raman molecules were used as signal probes. The SERS intensity of the core-satellite structure was associated with the number of satellite Ag nanoparticles around the core Au nanorods. When the target protein was added, both the protein and DNA could bond with aptamer. The competition between them led to changes in the Raman signal intensity, which could be used to determine the target protein and forecast its content. Herein, the release of the core-satellite assemblies occurred because of the high specific biorecognition of aptamer and Mucin-1. In order to evaluate the sensitivity of this system, different Mucin-1 concentrations were utilized to detect the SERS spectra of core-satellite structures. The method succeeded in determining Mucin-1, with a detection limit of 4.3 aM [78]. Furthermore, He et al. optimized the signal probe by embedding the Raman molecular tags into nanoparticles covered with chitosan [79]. In this study, one magnetic chitosan modified with aptamer (or antibody) was used as a capture probe, based on the affinity binding site of the protein. The other silver/chitosan nanoparticles, modified with aptamer and encapsulated by Raman report molecules, were utilized as SERS sensing probes via the other binding site of the protein. The sandwich complexes of aptamer/protein/aptamer were formed, and the protein concentration could be detected by the intensity variation of the SERS signal of the Raman report molecules. After optimization, the LOD of the platelet-derived growth factor BB was as low as 3.2 pg/mL. The optimized signal probe could avoid the dissolution of the Raman tag molecules from the nanoparticles and increased the stability of the signal molecules. Compared with the conventional ELISA method, this aptamer recognition-induced target-bridged strategy exhibited a wider linear range, lower cost and more convenient operation. Additionally, SERS combined with the imaging technique possessed the ability to locate the lesion site and provided guides on tumor resection [80]. SERS was applied to monitor urine samples of subjects diagnosed with prostate cancer and healthy controls [81]. LDA is a classification method that uses one linear function to discriminate between the classes. By using PCA and LDA of the spectral data, the obtained classification model exhibited a high sensitivity of 100% and specificity of 89%. The results indicated that diagnostics based on urine SERS could discriminate prostate cancer from controls. In conclusion, SERS combined with the aptamer/immune recognition method can achieve the effective, sensitive detection of proteins and has the potential for clinical diagnosis (see Table 2).

## 4. SERS for Drug Detection

Drugs present specific biological and physiological effects on bodies and are used in the prevention, diagnosis, treatment and cure of diseases. Poison capsule events result from a lack of effective detection. Therefore, reasonable and effective tests have important implications in drug safety and human health. As a new means of detection, SERS has been successfully used in detecting the pesticide content in fruit, quantifying the drug crystals, active ingredients and accessories, tracking the drug release process in living cells [82,83,84]. This article will summarize the applications of SERS in drug detection in detail.

### 4.1. SERS in the Detection of Illegally Added Drugs for Drug Safety

In recent years, much more attention has been paid to Traditional Chinese Medicines (TCMs), which play an important role in medicine [85,86,87,88]. In order to gain more profits and quickly improve the efficacy of drugs, some manufactures may add some chemicals to drugs illegally. This behavior may do some harm to people who take the drugs. However, the phenomenon is becoming more common recently because of the lack of an evaluation standard and market-governance. It is important to detect the medicated additive, but many problems need to be solved, such as the complex pretreatment process, time-consuming steps and other issues. It is urgent to establish a reasonable and effective method to detect the illegal addition. Penicillin and its degradation product, 5-fluorouracil, have been successfully distinguished by SERS [89,90,91]. This proved that SERS and SERS combined separating technology have the potential to detect illegal drug addiction in guiding the establishment of a drug quality standard due to the low requirement of SERS on the purity of analytes.

#### 4.1.1. Direct Detection Method

Direct detection method usually detects the sample by SERS, without any pretreatment. The basic process includes following steps: first, the drugs are detected by SERS, and the corresponding SERS spectrum is obtained in an appropriate condition. Second, the spectrum of suspicious illegal additives detected at the same condition is evaluated. Finally, the illegally added ingredients are identified according to the overlapping of characteristic peaks. We used SERS to detect the successfully and illegally added drugs in Chinese Traditional Patent Medicines (CTPMs), such as Jiangtangshu capsules [92]. Herein, silver colloidal used as a SERS substrate was prepared by a sodium citrate reaction. After the optimization of silver colloidal aggregation and pH conditions, different chemicals in CTPM could be detected simultaneously. Konjac pressed candy (KPC), a natural slimming product (NSP), is used in the treatment of obesity. Sibutramine (SIB) is one of the most commonly adulterated anorexic medicines found in NSP. SERS coupled with chemometrics was used for the rapid discrimination and detection of SIB and its analogues (monodesmethyl- sibutramine, MDS; didesmethylsibutramine, DDS) in KPC. The LODs of SIB, MDS and DDS were 5 × 10^−8^ M, 5 × 10^−7^ M and 10^−6^ M, respectively [93]. The direct determination method is simple, time-saving, inexpensive and easy to operate. However, the complicated composition of TCMs usually induces background interference and spectral overlap and reduces the accuracy of detection. 

#### 4.1.2. SERS Combined with Separation Technology

The combination of SERS and separation technology means that SERS is used for detection after a separation process. This method can significantly reduce background interference and increase the accuracy of the results. TLC-SERS, the most common combination of technologies, has been utilized for the detection of dye in the art and pollution of aromatic hydrocarbons in water [94,95]. TLC-SERS has also been used to detect illegal adulterants in diet pills and plant dietary supplements. Due to the uncontrollable stimulating side effects, adulteration of ephedrine and its analogue in botanical dietary supplements (BDS) is prohibited. To directly identify trace adulterants, TLC was utilized to separate four analogues prior to SERS detection [96]. Herein, ephedrine and its analogues, including norephedrine, pseudoephedrine, and methylephedrine, were mixed, and the mixture was used as the sample. The results indicated that the obtained TLC-SERS method was able to recognize these four analogues, and eight common Raman peaks were extracted to establish the reference-free detection model. Lu et al. also applied the TLC-SERS strategy to detect anti-diabetes chemicals used to adulterate BDS for diabetes [97]. Under optimized experimental conditions, the highly sensitive detection of 0.001% (w/w) adulteration could be achieved. In addition, chemicals in extremely complex herbal matrices could also be identified by the TLC-SERS method. Lu et al. applied the TLC to separate adulterants in BDS [98]. Then, dynamic surface enhanced Raman spectroscopy (DSERS) detection was performed with a portable Raman spectrometer. It was found that a higher SERS enhancement and stability were obtained, because 50% glycerol Ag colloid was chosen as the active substrate. During the detection process, a large number of hot spots could be formed, and the target molecules were automatically concentrated. This method displayed a desirable stability, improved sensitivity, and could achieve separation and detection rapidly. To analyze real BDS samples, one sample adulterated with benproperine phosphate was detected. Compared with the TLC-SERS technique, the sensitivity has been improved by 1-2 orders of magnitude by using the TLC-DSERS technique, based on the increased hot spots. The combination of SERS and other technologies is one of the important development directions in SERS research.

### 4.2. SERS in the Detection of Drugs in Bodily Fluid for In Vivo Illegal Drugs Analysis

**Figure 6:**
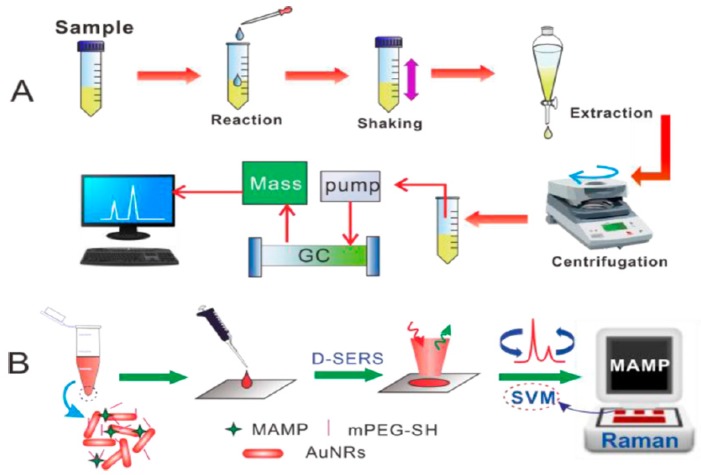
Comparison of drug detection in urine by (**A**) a standard procedure versus (**B**) the D-SERS and support vector machines (SVM) solution (Dong. et al. [100]).

As the most common body fluids, saliva and urine are easy to obtain and sample extraction causes little damage to the testee. The main ingredient in them is water, which does not significantly interfere with the results. They are suitable for detecting illegal drugs in the body, and a direct test is easy to operate. Han et al. applied a portable kit for the rapid SERS detection of drugs in human urine [99]. A 3 min pretreatment for the separation of amphetamines from human urine was utilized. An ultraperformance liquid chromatography (UPLC) examination indicated that the proposed pretreatment procedure was able to lower the high background signals of complex components in urine. Using the substrate of 2D gold nanorod (GNR) arrays, methamphetamine (MA), 3,4-methylenedioxymethamphetamine (MDMA) and methcathinone (MC) in volunteers’ urine samples, with various clinical natures, were measured. Herein, thirty batches of GNR arrays could generate an intensity of 1001 cm^−1^ in MA molecules, with an RSD of 7.9%. The LOD of amphetamines in human urine was 0.1 ppm. Dong et al. successfully detected and directly read MDMA in human urine using DSERS, with a portable Raman spectrometer on GNRs and a classification algorithm, called support vector machines (SVM) (Figure 6) [100]. In this study, enhanced SERS signals were obtained using DSERS, and then the SVM model was developed by choosing these data for fast identified and visual results. One of the main advantages of this system was that the detection results were displayed directly, without the need for an analysis of the spectra. The samples mixed with the colloidal sol of GNRs could be applied without pretreatment, and the drugs were detected from a wet state to a drying state. In comparison with the traditional method, in the lab, this method only consumed a 2 μL sample volume and took less than 2 min for detection. This method is not suitable for drugs associated with the SVM model, but it still has a satisfactory ability in conveniently and rapidly testing drugs on-site for the police.

### 4.3. SERS in the Detection of Drug Concentration for Medication Guides

Therapeutic drug monitoring is important for providing a clinical personalized treatment guide, reducing side effects and improving drug resistance. The typical methods for detecting drug concentration include: (1) HPLC, GC, which are suitable for the vast majority of drugs; (2) the immune method, which is used for protein and polypeptide drugs; (3) the microbial method, which is used for antibiotic compounds. These methods are time-consuming and relatively expensive. SERS, as a type of simple method with a high efficiency and sensitivity, shows a promising application prospect in nondestructive testing of the content of active ingredients and illegal drugs in blood. A silver colloid was selected as the SERS active substrate for detecting pethidine hydrochloride agents [101]. Herein, the SERS improvement efficiency of distinct substrate aggregates was explored to achieve optimal experimental conditions. The LOD for pethidine hydrochloride in water was 0.1 μg mL^−1^, which was lower than the typical administered dosages. Moreover, a favorable linear relationship between drug concentration and the Raman intensity was observed for pethidine hydrochloride at a concentration range of 0.1 to 10 μg mL^−1^. Self-assembled Au@Ag nanorod dimers were utilized to develop SERS substrates [102]. Benefiting from the enhanced electronic field after adding silver shell coating on an Au nanorod dimer surface, ultrasensitive dopamine detection could be achieved, with an LOD of 0.006 pM. SERS, combined with the new SERS substrate or other separation technologies, is also used to detect the blood drug concentration.

**Figure 7:**
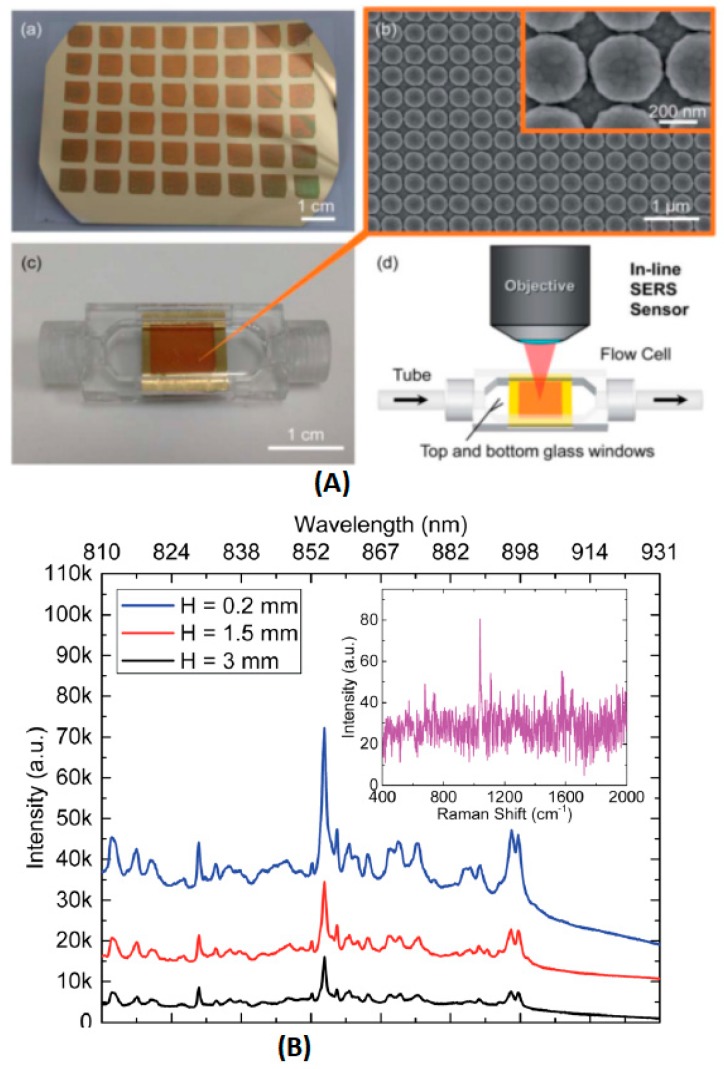
(**A**) (**a**) Image of the PNA surface fabricated on a flexible polymer sheet. (**b**) SEM image of the completed PNA surface. Inset: close-up SEM image of the PNA surface, with a measured inter-dome separation distance of 15-20 nm. (**c**) Image of the SERS sensor, assembled by the incorporation of the flexible PNA substrate into a plastic flow cell. (**d**) Schematic of an in-line SERS sensor, where two cylindrical openings at the ends of the SERS sensor are connected in a series with biomedical tubing and a 785 nm laser, focused on the PNA surface by a 50 objective. (**B**) SERS spectra of 25 mg mL^−1^ promethazine solution for SERS sensors with varying chamber heights (H). Primary SERS intensity peak for promethazine can be observed at 1037 cm^−1^. The inset represents the Raman spectrum of 25 mg mL^−1^ promethazine solution, acquired when the laser was focused on a uniform non-nanostructured gold surface. (by H. Y. Wu [103]).

Cunningham et al. utilized a plasmonic nanodome array (PNA) surface as an integrated SERS sensor for the point-of-care detection and real-time monitoring of intravenously delivered drugs via tubing (Figure 7) [103].

In this study, the PNA structures were constructed using a nanoreplica molding process. Gold was selected as the plasmonic material, because it was less susceptible than silver to oxidation. This sensor was then incorporated into a miniature flow cell that was connected in a series with intravenous drug delivery tubing. Ten pharmaceutical compounds were chosen as the model for SERS detection. The LODs of hydrocodone, levorphanol, and mitoxantrone were low in ng/mL and well below the typical administered dosages (mg/mL). This method also presented a desirable result in the co-detection of multiple drugs, with a high reproducibility and stability for at least five days after the drugs were administered. McLaughlin et al. applied surface-enhanced resonance Raman scattering (SERRS) to test for mitoxantrone in blood samples [104]. A simple, sensitive and fast method was developed by employing a flow cell and silver colloid, as the substrate. The LOD was as low as 0.02 ng/mL, which was close to the results of HPLC. Huang et al. used microwave-treated gold film-polyethylene beads as SERS substrates to detect the anti-cancer drug (paclitaxel) concentration in blood plasma [105]. The representative Raman peak (1605 cm^−1^) intensity of paclitaxel was selected to estimate the paclitaxel concentration after deducting the Raman background from the blood plasma. A satisfactory linear relation was observed from 10^−8^ M to 10^−7^ M. This indicated that microwave-modified Au-PS SERS substrates had a promising capacity to quickly and efficiently monitor the anti-cancer drug distribution in blood plasma.

**Figure 8:**
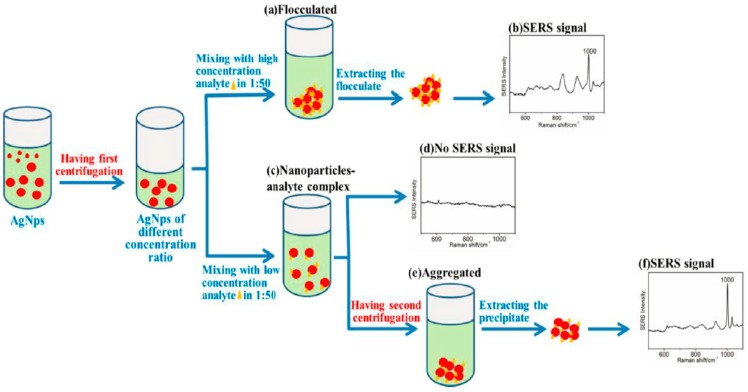
Diagram of the detection process of phenformin hydrochloride by the SERS-based two-step centrifugation method. (**a**) The concentrated AgNPs were flocculated when high concentration phenformin hydrochloride was added. (**b**) Characteristic peak of the phenformin hydrochloride were detected at 1000 cm^−1^. (**c**) The concentrated AgNPs were unflocculated, and an analyte-AgNPs complex was formed when a low concentration analyte was added. (**d**) No characteristic peak of the phenformin hydrochloride was detected. (**e**) An aggregated analyte-AgNPs complex and the interparticle gap decreased after the second centrifugation. (**f**) Characteristic peak of the phenformin hydrochloride were obvious at 1000 cm^−1^ (Chen et al. [106]).

Blood contains a lot of information, and SERS has the potential for low concentration detection due to its high sensitivity, although there are still some problems that need to be solved in detecting the drug concentration in blood. For example, the strong interference of blood, the poor reproducibility of the sol matrix, and last but not least, the low drug concentration in the body. AgNPs was prepared by a hydroxylamine hydrochloride reaction as an SERS substrate, and we used an SERS-based two-step centrifugation method to detect phenformin hydrochloride, with a detection limit of 500 fM, which was about 10^−5^ times higher than that of other conventional methods [106] (Figure 8). 

This method included two steps: (1) centrifuging colloidal silver to concentrate it and increasing the chance of analyte adsorption on nanoparticles; (2) centrifuging samples after the interest was mixed with nanoparticles to increase the formation of hot-spot. This method was easy to operate and produced a greatly improved sensitivity. The two-step centrifugation method had a great capacity for drug blood concentration detection. Since drug blood concentration detection is the focus of research at present and even in the future, efforts should be made to improve the method for accurately detecting drug blood concentration in real samples. For cancer treatment, the ultrasensitive detection of low-quantity drugs is essential for personalized therapy. SERS provides a useful method for precisely identifying analytes based on the specific vibrational spectra. Four anticancer drugs, including sunitinib, paclitaxel, irinotecan and SN-38, were detected by SERS [107]. PCA was performed with a built-in routine, running under MatLab R2012a. The results suggested that low concentrations of sunitinib, irinotecan and SN-38 could be detected by SERS, with a 633 nm laser excitation. The three drugs all exhibited a linearity range of 10^2^–10^3^ ng. Irinotecan and SN-38 possessed similar Raman fingerprint due to their similar molecular structure, although the multivariate analysis combined with Raman spectra facilitated the discrimination of these two drugs. This study developed an efficient SERS-based technique for monitoring chemotherapy drugs in the cancer therapy process.

## 5. Conclusions and Perspectives

This review focuses on the innovative SERS-based strategies for detecting DNA, protein and drugs. As an important modern spectroscopic technique, SERS has attracted tremendous attention in bio-science due to its high-sensitivity, high-selectivity, the noninterference of water and non-destructive testing of analytes. 

Various methods, including the DNA hybridization method, asymmetry signal amplification method, and gene chips method, present unique benefits in the detection of disease-related DNA targets for clinical diagnosis. Moreover, the sandwich detection and amplification methods also provide efficient protocols for detecting microbe-related DNA targets for food safety. Compared with conventional microbial monitoring, the SERS-based strategy is a time-saving technique for detecting gene sequences, with a high accuracy and specificity. Since proteins are important cancer biomarkers, SERS utilized for detecting proteins plays an important role in clinical diagnosis. Furthermore, multivariate data analysis associated with SERS facilitates the recognition and classification of complicated spectral data. The combination of the separation technique and SERS has gained much research interest in detecting illegally added drugs. To measure drug concentrations for medication guides, multiplex detection based on SERS displays a promising prospect. 

With the in-depth research on developing materials for the construction of active SERS substrates, the combination of technologies, and the establishment of the Raman spectra database, we foresee a promising potential in the application of SERS for DNA, protein and drug detection. However, despite its tremendous potential, it is still necessary to enhance the analytical performances of SERS. The challenge of this technique is to develop a standardized method for the formation of homogeneous SERS covering on targets. Moreover, considerable efforts will continue to be made to achieve quantitative detecting analytes in complex biological samples.

## Figures and Tables

**Table 1:** SERS for DNA detection.

Substrate	Analyte	Detection Limit	Detection Method	Ref.
PSA/Ag-NP composite nanosphere	4-Aminothiophenol	10^−9^ M	Sandwich detection method	[35]
Gold nanoparticle	*S. typhimurium, S. aureus*	15 fM	Sandwich detection method	[36]
Gold nanoparticle	Target DNA	50 pM	Amplification method	[39]
Gold nanorod	Target oligonucleotide sequence	6.3 fM	Amplification method	[40]
Gold nanoparticle	DNA methylation	3 pM	Asymmetry signal amplification method	[49]
Gold nanoparticle	MicroRNA, ATP	0.15 fM	Asymmetry signal amplification method	[50]
Gold nanoparticle	Target DNA	10 pM	Gene chips method	[51]

**Table 2:** SERS for protein detection.

Substrate	Application	Year	Ref.
Silver nanoparticle	Bluminal A breast cancer detection	2015	[68]
Silver nanoparticle	Nasopharyngeal cancer detection	2010	[69]
Silver nanoparticle	Noninvasive cancer detection	2011	[70]
Gold nanoparticle	Cancer diagnostic immunoassay	2013	[75]
Gold nanoparticle	Serological liver cancer biomarkers detection	2014	[77]
Silver nanoparticle	Mucin-1 detection	2015	[78]
Silver nanoparticle	Protein detection	2015	[79]
